# Mortality and Cause of Death Among Youths Previously Incarcerated in the Juvenile Legal System

**DOI:** 10.1001/jamanetworkopen.2021.40352

**Published:** 2021-12-23

**Authors:** Donna A. Ruch, Danielle L. Steelesmith, Guy Brock, Samantha J. Boch, Camille R. Quinn, Jeffrey A. Bridge, John V. Campo, Cynthia A. Fontanella

**Affiliations:** 1 Center for Suicide Prevention and Research, Abigail Wexner Research Institute at Nationwide Children’s Hospital, Columbus, Ohio; 2 Department of Psychiatry and Behavioral Health, The Ohio State University Wexner Medical Center, Columbus; 3 Department of Biomedical Informatics, The Ohio State University College of Medicine, Columbus; 4 Cincinnati Children’s Hospital Medical Center, University of Cincinnati, Cincinnati, Ohio; 5 The College of Social Work, The Ohio State University, Columbus; 6 Department of Pediatrics, The Ohio State University College of Medicine, Columbus; 7 Department of Psychiatry and Behavioral Sciences, Johns Hopkins University School of Medicine, Baltimore, Maryland

## Abstract

**Importance:**

More than 50 000 youths are incarcerated in the United States on any given day, and youth incarceration has been linked to lasting adverse outcomes, including early mortality. Improving our understanding of the factors associated with early mortality among incarcerated youths can inform appropriate prevention strategies.

**Objective:**

To examine mortality rates and causes of death among youths previously incarcerated in the juvenile legal system.

**Design, Setting, and Participants:**

This retrospective longitudinal population-based cohort study compared mortality rates between youths aged 11 to 21 years incarcerated from 2010 to 2017 with same-aged nonincarcerated Medicaid-enrolled youths in the state of Ohio. Data from January 2017 to December 2019 were collected from juvenile incarceration, Medicaid, and death certificate information in Ohio.

**Exposure:**

Incarceration in the state of Ohio’s juvenile legal system.

**Main Outcomes and Measures:**

Number, characteristics, and causes of deaths. Poisson regression incidence rate ratios (IRRs) compared mortality rates between previously incarcerated and Medicaid-enrolled youths.

**Results:**

Among 3645 incarcerated youths, 3398 (93.2%) were male, 2155 (59.1%) Black, 1307 (35.9%) White, and 183 (5.0%) other race and ethnicity. Overall, 113 youths (3.1%) died during the study period. Homicide was the leading cause of death in formerly incarcerated youths (homicide: 63 [55.8%]; legal intervention [ie, death due to injuries inflicted by law enforcement]: 3 [2.7%]). All-cause mortality rates were significantly higher among previously incarcerated youths than Medicaid-enrolled youths (adjusted IRR [aIRR], 5.91; 95% CI, 4.90-7.13) in every demographic subgroup. Compared with Medicaid-enrolled youths, mortality rates for previously incarcerated youths were highest for homicide (aIRR, 11.02; 95% CI, 8.54-14.22), overdose (aIRR, 4.32; 95% CI, 2.59-7.20), and suicide (aIRR, 4.30; 95% CI, 2.22-8.33). Formerly incarcerated Black youths had a significantly higher risk of homicide (aIRR, 14.24; 95% CI, 4.45-45.63) but a lower risk of suicide (aIRR, 0.18; 95% CI, 0.04-0.89) and overdose (aIRR, 0.31; 95% CI, 0.10-0.99) than White youths who were incarcerated. Previously incarcerated youths aged 15 to 21 years were significantly more likely to die than youths aged 22 to 29 years, irrespective of cause of death (aIRR for youths aged 22-29 years, 0.09; 95% CI, 0.06-0.14).

**Conclusions and Relevance:**

In this study, youths with a history of incarceration were significantly more likely to experience early mortality compared with nonincarcerated Medicaid-enrolled youths. Delinquency and violence prevention strategies that incorporate a culturally informed approach and consider sex and developmental level are critical.

## Key Points

**Question:**

Are youths with a history of incarceration at increased risk of early mortality compared with youths with no history of incarceration?

**Findings:**

In this cohort study of 3645 previously incarcerated youths, the all-cause mortality rate was 5.9 times higher in previously incarcerated youths than the rate observed in general population, Medicaid-enrolled youths. Homicide was the leading cause of death among formerly incarcerated youths, accounting for more deaths than all other causes combined.

**Meaning:**

These findings suggest that delinquency and violence prevention strategies that incorporate a culturally informed approach and consider sex and developmental level are critical to reduce early mortality in this high-risk youth population.

## Teaser

This cohort study examines mortality rates and causes of death among youths previously incarcerated in the juvenile legal system.

## Introduction

Nearly 50 000 youths are confined in juvenile correctional facilities on any given day in the United States.^1^ The US juvenile legal system was built on a rehabilitative conviction and designed to focus on the distinct needs of youths rather than to impose punitive sanctions as in the adult criminal system.^2^ In spite of this reformative foundation, youth incarceration has been associated with lasting adverse outcomes including academic failure, limited vocational opportunities, poor physical and mental health, and a lifetime of criminal behavior.^3,4,5^ Evidence also suggests incarcerated youths may be at elevated risk of early mortality compared with youths in the general population.^6,7,8,9^ This is especially disconcerting for Black youths, who experience disproportionately higher rates of confinement compared with other racial/ethnic groups.^1^

Research examining mortality rates in youths involved with the juvenile legal system has reported consistent results.^6,7,8,9^ In one study, youths aged 15 to 22 years released from detention were 5 times more likely to die during 7 years of follow-up compared with youths in the general population, with 75% of deaths among detained youths attributed to homicide.^6^ Aalsma et al^7^ examined mortality in youths aged 10 to 18 years along the continuum of system involvement from arrest to incarceration and found the mortality rate for incarcerated youths was 2.5 times higher than that among youths who were arrested, but not detained. Approximately half of all deaths (48%) were homicides.^7^ Teplin et al^8^ followed up youths for as long as 16 years after detention and found mortality rates of detained youths were 4 times that of the general population, with 68% of deaths by homicide.^8^

Violence prevention and homicide reduction are leading health indicators for the US Department of Health and Human Services’ Healthy People 2030 initiative, with a specific goal to decrease youth fatalities from violence.^10^ Earlier research has focused on mortality in youths detained short term in a single county-level juvenile detention center^6,7,8,9^; however, less attention has been given to mortality in youths receiving lengthier incarceration sentences. Using statewide data from multiple long-term juvenile correctional facilities, the current study addresses this gap by examining mortality among previously incarcerated youths compared with Medicaid-enrolled youths in the general population. Findings provide updated evidence to inform delinquency and violence prevention strategies to reduce early death in this vulnerable youth population.

## Methods

Using an integrated database that links data from multiple youth-serving systems, this retrospective longitudinal population-based cohort study compared mortality rates between youths aged 11 to 21 years who were previously incarcerated in a juvenile correctional facility in Ohio between January 1, 2010, and December 31, 2017, with same-aged nonincarcerated youths who were enrolled in Medicaid for at least 30 continuous days during the study period. Of the 3813 previously incarcerated youths identified for the study period, 168 privately insured or uninsured youths had no history of Medicaid enrollment and were excluded from the analyses based on unavailable data. Informed consent was waived for this study, which was considered exempt according to the review policies of the Abigail Wexner Research Institute at Nationwide Children’s Hospital. This study followed the Strengthening the Reporting of Observational Studies in Epidemiology (STROBE) guidelines for observational studies.^11^

The final sample included 3645 previously incarcerated and 1 171 260 Medicaid-enrolled youths. Death certificate data were obtained from Ohio’s Department of Vital Statistics to identify number of deaths based on *International Classification of Diseases and Related Health Problems, Tenth Revision *(*ICD-10*), codes for cause of death.^12^ Juvenile incarceration, Medicaid, and death certificate data were merged based on an algorithm used in earlier studies that incorporates social security numbers, date of birth, and sex.^13,14^ These data were collected from January 2017 to December 2019.

### Measures

Cause of death based on *ICD-10* codes was categorized as homicide (including legal intervention [ie, death due to injuries inflicted by law enforcement]), suicide, overdose, and other (eTable in the Supplement).^12^ Race and ethnicity (Black, White, and other [American Indian or Alaska Native, Asian or Pacific Islander, Hispanic, multiracial, and other or unknown]) was defined based on participant self-reporting and Medicaid classifications^15^ and included in the analyses as possible confounders associated with mortality outcomes. Age at time of death or end of study period as well as age at release from incarceration were also captured. Offense type was the most severe charge resulting in incarceration and categorized as person (eg, assault, robbery, murder, rape), property (eg, arson, burglary), or other (eg, public order, drug, complicity, gang activity, and retaliation). A variable for length of incarceration was created based on the total time an individual was confined during the study period. Youths were identified as repeat offenders if they had 2 or more periods of incarceration during the study period.

### Statistical Analysis

The number of previously incarcerated decedents was determined by cause of death and stratified by demographic and incarceration-related characteristics. Mortality rates per 100 000 persons were calculated for previously incarcerated and Medicaid-enrolled youths overall and by demographic characteristics and causes of death. Poisson regression was used to calculate incidence rate ratios (IRRs) comparing rates of death between groups, adjusting for age, sex, and race and ethnicity. Similar regression analyses examined adjusted IRRs (aIRRs) comparing rates of death among previously incarcerated decedents. The Wald method was used to calculate 95% CIs. Cumulative event probabilities for each outcome were estimated using cumulative incidence curves, which account for the occurrence of other competing events. Cumulative incidence curves were estimated for previously incarcerated and Medicaid-enrolled youths as well as by race and ethnicity among previously incarcerated youths. Rates and IRRs were calculated using SAS version 9.4 (SAS Institute), and event probabilities were calculated in R version 3.6 (R Project for Statistical Computing) with the compress package. Statistical significance was set at *P* < .05, and all tests were 2-tailed.

## Results

### Characteristics of Previously Incarcerated Youth

Among 3645 previously incarcerated youth, 3398 (93.2%) were male, 2155 (59.1%) Black, 1307 (35.9%) White, and 183 (5.0%) other race and ethnicity (Table 1). There were 1145 youths (31.5%) aged 15 to 21 years at time of death or study end, and 2496 (68.5%) aged 22 to 29 years. Slightly more than half of incarcerated youths (1895 [52.0%]) were aged 18 to 21 years when released from confinement. The predominant offense types were person (2225 [61.0%]) and property (919 [25.2%]). Most youths were incarcerated for less than or equal to 1 year (2145 [58.8%]) and not repeat offenders (3040 [83.4%]).

**Table 1.** Characteristics and Cause of Death Among Youths Previously Incarcerated in the Juvenile Legal System, 2010 to 2017, With Death Data Through 2019

Characteristic	Youth, No. (%)					
	Total (N = 3645)	All cause (n = 113)	Homicide (n = 65)	Suicide (n = 9)	Overdose (n = 15)	Other (n = 24)^a^
Sex						
Female	247 (6.8)	6 (5.3)	3 (4.6)	0	2 (13.3)	1 (4.2)
Male	3398 (93.2)	107 (94.7)	62 (95.4)	9 (100.0)	13 (86.7)	23 (95.8)
Race and ethnicity						
Black	2155 (59.1)	80 (70.8)	61 (93.8)	2 (22.2)	4 (26.7)	13 (54.2
White	1307 (35.9)	29 (25.7)	3 (4.6)	7 (77.8)	11 (73.3)	8 (33.3)
Other^b^	183 (5.0)	4 (3.5)	1 (1.5)	0	0	3 (12.5)
Age at death or study end, y						
15-21	1149 (31.5)	69 (61.1)	42 (64.6)	5 (55.6)	6 (40.0)	16 (66.7)
25-29	2496 (68.5)	44 (38.9)	23 (35.4)	4 (44.4)	9 (60.0)	8 (33.3)
Age at release, y						
11-17	1750 (48.0)	52 (46.0)	32 (49.2)	4 (44.4)	8 (53.3)	8 (33.3)
18-21	1895 (52.0)	61 (54.0)	33 (50.8)	5 (55.6)	7 (46.7)	16 (66.7)
Offense type						
Person	2225 (61.0)	59 (52.2)	33 (50.8)	6 (66.7)	6 (40.0)	14 (58.3)
Property	919 (25.2)	33 (29.2)	18 (27.7)	2 (22.2)	8 (53.3)	5 (20.8)
Other^c^	501 (13.7)	21 (18.6)	14 (21.5)	1 (11.1)	1 (6.7)	5 (20.8)
Length of incarceration, y						
≤1	2145 (58.8)	71 (62.8)	39 (60.0)	6 (66.7)	13 (86.7)	13 (54.2)
>1	1500 (41.2)	42 (37.2)	26 (40.0)	3 (33.3)	2 (13.3)	11 (45.8)
Repeat offender						
No	3040 (83.4)	96 (85.0)	56 (86.2)	7 (77.8)	10 (66.7)	23 (95.8)
Yes	605 (16.6)	17 (15.0)	9 (13.8)	2 (22.2)	5 (33.3)	1 (4.2)

^a^ Other cause of death includes natural causes (11 youth); accident (7 youth); and other or unknown (6 youth).

^b^ Other race and ethnicity includes Asian/Pacific Islander (7 youth); Hispanic (78 youth); multiracial (52 youth); American Indian or Alaska Native (8 youth); and other or unknown (38 youth).

^c^ Other offense type includes drug-related (139 youth); public-order crimes (322 youth); and other or unknown (40 youth).

There were 113 previously incarcerated youths (3.1%) who died during the study period. Homicide was the leading cause of death (homicide: 63 [55.8%]; legal intervention: 3 [2.7%]) followed by overdose (15 [13.3%]). Most decedents were male (107 [94.7%]), Black (80 [70.8%]), aged 15 to 21 years (69 [61.1%]), and aged 18 to 21 years when released from confinement (61 [54.0%]). Most youths who died were charged with a person offense (59 [52.2%]), incarcerated for less than or equal to 1 year (71 [62.8%]), and not repeat offenders (96 [85.0%]). Homicide was the most common cause of death regardless of sex, age, age at release, offense type, length of incarceration, or repeat offender status. More White youths died by overdose than any other cause of death, while more Black youths died by homicide.

### Mortality Rates Between Previously Incarcerated and Medicaid-Enrolled Youth

The all-cause mortality rate for youths with a history of incarceration was significantly higher than Medicaid-enrolled youths (aIRR, 5.91; 95% CI, 4.90-7.13), which was a consistent finding in every demographic subgroup (Table 2). Mortality rates for each specific cause of death were also higher in previously incarcerated youths compared with Medicaid-enrolled youths, with the largest difference observed for all youths in deaths by homicide (aIRR, 11.02; 95% CI, 8.54-14.22), followed by overdose (aIRR, 4.32; 95% CI, 2.59-7.20), and suicide (aIRR, 4.30; 95% CI, 2.22-8.33). Among males, mortality rates were highest in deaths by homicide (aIRR, 10.16; 95% CI, 7.82-13.19) and suicide (aIRR, 4.18; 95% CI, 2.16-8.10). Mortality rates for previously incarcerated females were significantly higher than Medicaid-enrolled youths for homicide (aIRR, 41.12; 95% CI, 13.11-128.98) and overdose (aIRR, 10.84; 95% CI, 2.70-43.38). No formerly incarcerated females in the cohort died by suicide.

**Table 2.** Cause of Death Among Previously Incarcerated Youths in the Juvenile Legal System and Medicaid Comparison Group, 2010-2017, With Death Data Through 2019

Characteristic	Mortality rate (95% CI)		aIRR (95% CI)^a^
	Incarcerated	Medicaid	
**All cause**			
All	575.17 (478.32-691.62)	79.76 (77.94-81.63)	5.91 (4.90-7.13)
Sex			
Female	422.45 (189.79-940.33)	53.81 (51.73-55.97)	8.81 (3.95-19.64)
Male	587.07 (485.74-709.54)	106.80 (103.80-109.89)	5.56 (4.58-6.74)
Race and ethnicity			
Black	710.54 (570.71-884.61)	85.53 (82.04-89.17)	6.52 (5.20-8.18)
White	391.96 (272.38-564.04)	78.79 (76.55-81.10)	4.34 (3.01-6.26)
Other^b^	404.61 (151.86-1078.04)	64.14 (57.98- 70.94)	6.12 (2.27-16.48)
Age, y			
15-21	2371.85 (1873.33-3003.03)	89.96 (87.16-92.86)	20.02 (15.75-25.44)
22-29	262.89 (95.63-353.26)	70.68 (68.34-73.10)	2.80 (2.07-3.77)
**Homicide**			
All	330.85 (259.45-421.90)	14.26 (13.50 − 15.06)	11.02 (8.54-14.22)
Sex			
Female	211.23 (68.13-654.92)	4.99 (4.39-5.68)	41.12 (13.11-128.98)
Male	340.17 (265.21-436.31)	23.91 (22.52-25.40)	10.16 (7.82-13.19)
Race/ethnicity			
Black	541.78 (421.54-696.32)	36.31 (34.06-38.71)	10.95 (8.40-14.27)
White	40.55 (13.08-125.72)	4.46 (3.95-5.03)	8.64 (2.75-27.14)
Other^b^	101.15 (4.25-718.09)	14.93 (12.12-18.40)	5.96 (0.82-43.31)
Age, y			
15-21	1443.73 (1066.95-1953.58)	18.19 (16.95-19.52)	32.26 (23.58-44.12)
22-29	137.42 (91.32-206.79)	10.76 (9.87-11.73)	4.63 (3.04-7.05)
**Suicide**			
All	45.81 (23.84-88.04)	10.66 (10.01-11.36)	4.30 (2.22-8.33)
Sex			
Female	NA	5.12 (4.51-5.82)	NA
Male	49.38 (25.69-94.90)	16.43 (15.28 − 17.67)	4.18 (2.16-8.10)
Race/ethnicity			
Black	17.76 (4.44-71.03)	6.96 (6.01-8.05)	2.04 (0.50-8.28)
White	94.61 (45.10-198.46)	12.46 (11.59 − 13.40)	6.62 (3.14-13.99)
Other^b^	NA	8.99 (6.87-11.77)	NA
Age, y			
15-21	171.87 (71.54-412.93)	14.17 (13.08-15.35)	16.67 (4.41-25.80)
22-29	23.90 (8.97-63.68)	7.53 (6.80-8.35)	2.26 (0.84-6.07)
**Overdose**			
All	76.35 (46.03-126.65)	15.03 (14.26-15.86)	4.32 (2.59-7.20)
Sex			
Female	140.82 (35.22-563.05)	12.13 (11.16-13.18)	10.84 (2.70-43.48)
Male	71.33 (41.42-122.84)	18.06 (16.85-19.36)	4.00 (2.31-6.94)
Race/ethnicity			
Black	35.53 (13.33-94.66)	6.50 (5.58-7.56)	3.53 (1.30-9.61)
White	148.68 (82.34-268.46)	19.50 (18.40-20.66)	4.94 (2.72-8.98)
Other^b^	NA	8.14 (96.14-10.81)	NA
Age, y			
15-21	206.25 (92.66-459.08)	7.71 (6.92-8.59)	31.86 (14.13-71.85)
22-29	53.77 (27.98-103.35)	21.56 (20.28-22.92)	2.69 (1.39-5.19)
**Other^c^**			
All	122.16 (81.88-182.26)	39.81 (38.53-41.13)	3.22 (2.15-4.83)
Sex			
Female	70.41 (9.92-499.84)	31.57 (29.99-33.24)	2.69 (0.38-19.13)
Male	126.19 (83.86-189.90)	48.39 (46.39-50.49)	3.22 (2.13-4.87)
Race/ethnicity			
Black	115.46 (67.04-198.85)	35.77 (33.53-38.15)	2.95 (1.70-5.12)
White	108.13 (54.07-216.21)	42.38 (40.74-44.08)	2.64 (1.32-5.29)
Other^b^	32.07 (27.81-36.98)	303.46 (97.87-940.89)	11.60 (3.66-36.76)
Age, y			
15-21	549.99 (336.94-897.76)	49.89 (47.82-52.06)	10.20 (6.23-16.71)
22-29	47.80 (23.90-95.58)	30.83 (29.29-32.44)	1.31 (0.65-2.63)

Abbreviations: aIRR, adjusted incidence rate ratio, NA, not applicable.

^a^ IRRs adjusted for sex, race and ethnicity, and age.

^b^ Other race and ethnicity includes Asian/Pacific Islander, Hispanic, multiracial, American Indian or Alaska Native, and other or unknown.

^c^ Other cause of death include natural causes, accident, and other or unknown.

Black (aIRR, 10.95; 95% CI, 8.40-14.27) and White (aIRR, 8.64; 95% CI, 2.75-27.14) youths who were incarcerated had significantly higher mortality rates by homicide compared with Medicaid-enrolled youths. Formerly incarcerated White youths had significantly higher mortality rates for suicide (aIRR, 6.62; 95% CI, 3.14-13.99), and both Black and White youths were significantly more likely to die by overdose (Black youth: aIRR, 3.53; 95% CI, 1.30-9.61; White youth: aIRR, 4.94; 95% CI, 2.72-8.98) compared with Medicaid-enrolled youths. Mortality rates in youths who died between the ages of 15 and 21 years were significantly higher for previously incarcerated youths than Medicaid-enrolled youths regardless of cause of death, and by homicide and overdose for youths aged 22 to 29 years.

**Figure 1.**
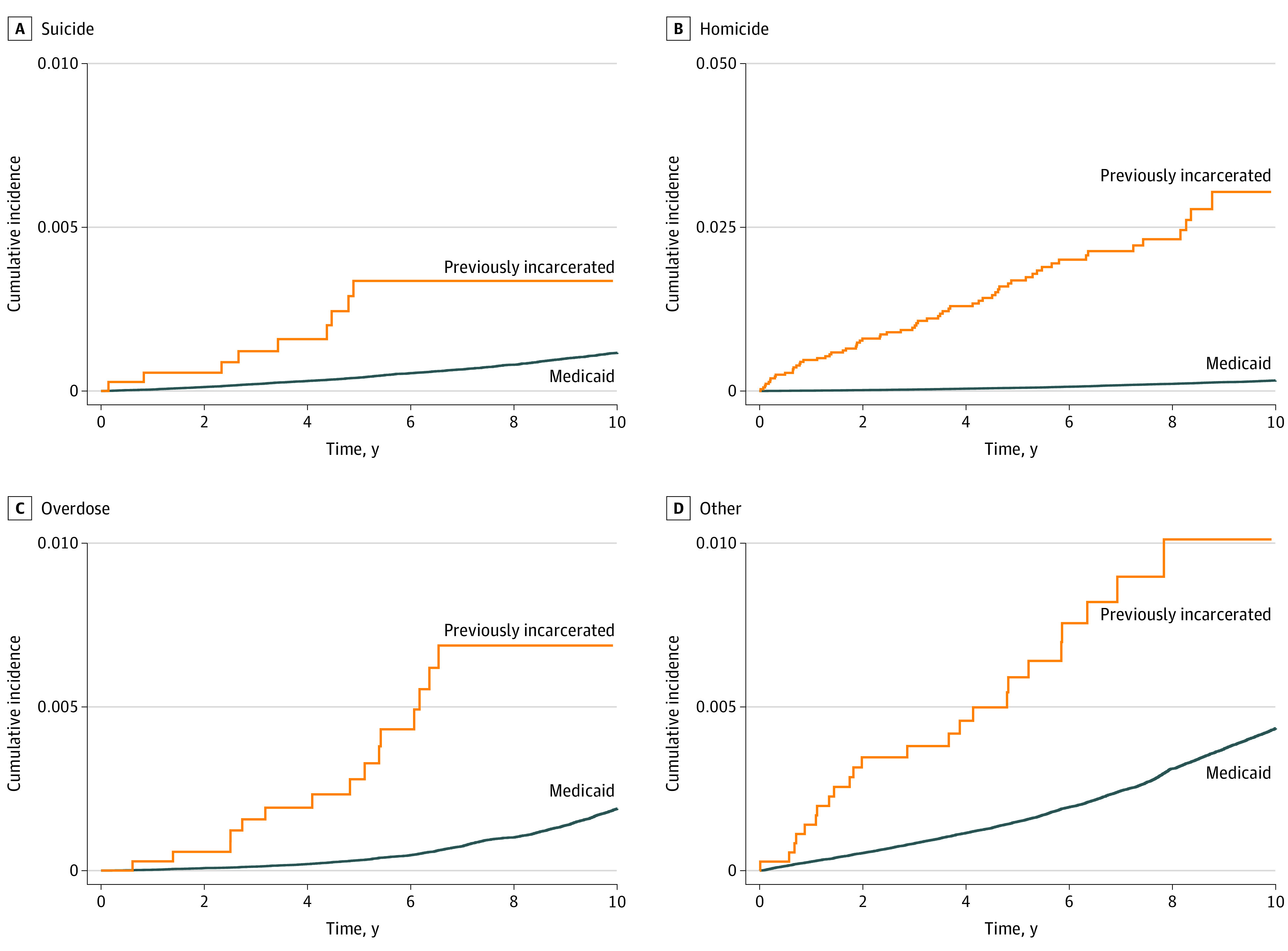
Cumulative Incidence by Cause of Death for Youths Previously Incarcerated in the Juvenile Legal System and Medicaid Comparison Group

Figure 1 displays the cumulative incidence over time by cause of death for previously incarcerated and Medicaid-enrolled youths. At 5 years, the cumulative incidence of suicide for youths who were incarcerated was 0.34%, more than 8 times greater than that for Medicaid-enrolled youths (0.04%). Increases of a similar order of magnitude were observed for 5-year cumulative incidence of homicide (1.7% vs 0.05%), accidental overdose (0.28% vs 0.03%), and other causes of death (0.59% vs 0.15%).

### Mortality Rates Among Previously Incarcerated Youth

Black youths were more likely to die than White youths overall, which was associated with higher rates of homicide (aIRR, 14.24; 95% CI, 4.45-45.63), and significantly less likely to die by suicide (aIRR, 0.18; 95% CI, 0.04-0.89) and overdose (aIRR, 0.31; 95% CI, 0.10-0.99) (Table 3). Youths aged 15 to 21 years were significantly more likely to die than youths aged 22 to 29 years, irrespective of cause of death (aIRR for youths aged 22-29 years, 0.09; 95% CI, 0.06-0.14), while youths released between the ages of 18 to 21 years were significantly more likely to die compared with youths released when aged 11 to 17 years (aIRR for youths released aged 18-21 years, 2.09; 95% CI, 1.41-3.11). Youths who were repeat offenders were significantly more likely to die by overdose compared with youths who were not repeat offenders (aIRR, 3.00; 95% CI, 1.01-8.92).

**Table 3.** Comparison of Characteristics by Cause of Death Among Youths Previously Incarcerated in the Juvenile Legal System, 2010 to 2017, With Death Data Through 2019

Characteristic	aIRR (95% CI) by cause of death				
	All cause	Homicide	Suicide	Overdose	Other^a^
Sex					
Female	1 [Reference]	1 [Reference]	NA	1 [Reference]	1 [Reference]
Male	1.32 (0.57-3.02)	1.43 (0.44-4.62)	NA	0.61 (0.14-2.71)	1.53 (0.20-11.50)
Race and ethnicity					
White	1 [Reference]	1 [Reference]	1 [Reference]	1 [Reference]	1 [Reference]
Black	1.91 (1.24-2.94)	14.24 (4.45-45.63)	0.18 (0.04-0.89)	0.31 (0.10-0.99)	0.95 (0.39-2.32)
Other^b^	1.15 (0.40-3.27)	2.85 (0.30-27.39)	NA	NA	3.00 (0.79-11.35)
Age at death or study end, y					
15-21	1 [Reference]	1 [Reference]	1 [Reference]	1 [Reference]	1 [Reference]
22-29	0.09 (0.06-0.14)	0.08 (0.05-0.14)	0.12 (0.03-0.46)	0.21 (0.07-0.62)	0.07 (0.03-0.16)
Age at release, y					
11-17	1 [Reference]	1 [Reference]	1 [Reference]	1 [Reference]	1 [Reference]
18-21	2.09 (1.41-3.11)	1.83 (1.09-3.07)	2.02 (0.49-8.26)	1.67 (0.57-4.88)	3.42 (1.39-8.45)
Offense type					
Person	1 [Reference]	1 [Reference]	1 [Reference]	1 [Reference]	1 [Reference]
Property	1.51 (0.97-2.36)	1.80 (0.99-3.28)	0.56 (0.11-2.88)	1.91 (0.64-5.69)	1.10 (0.38-3.20)
Other^c^	1.41 (0.85-2.35)	1.75 (0.92-3.33)	0.58 (0.07-4.96)	0.50 (0.06-4.23)	1.59 (0.55-4.59)
Length of incarceration, y					
≤1	1 [Reference]	1 [Reference]	1 [Reference]	1 [Reference]	1 [Reference]
>1	0.85 (0.56-1.30)	0.91 (0.53-1.57)	0.74 (0.17-3.26)	0.28 (0.06-1.36)	1.23 (0.51-3.01)
Repeat offender					
No	1 [Reference]	1 [Reference]	1 [Reference]	1 [Reference]	1 [Reference]
Yes	0.98 (0.58-1.67)	0.91 (0.44-1.88)	1.61 (0.33-7.97)	3.00 (1.01-8.92)	0.21 (0.03-1.59)

Abbreviations: aIRR, adjusted incidence rate ratio; NA, not applicable.

^a^ Other cause of death includes natural causes, accident, and other or unknown.

^b^ Other race and ethnicity includes Asian/Pacific Islander, Hispanic, multiracial, American Indian or Alaska Native, and other or unknown.

^c^ Other offense type includes drug-related, public-order crimes, and other or unknown.

**Figure 2.**
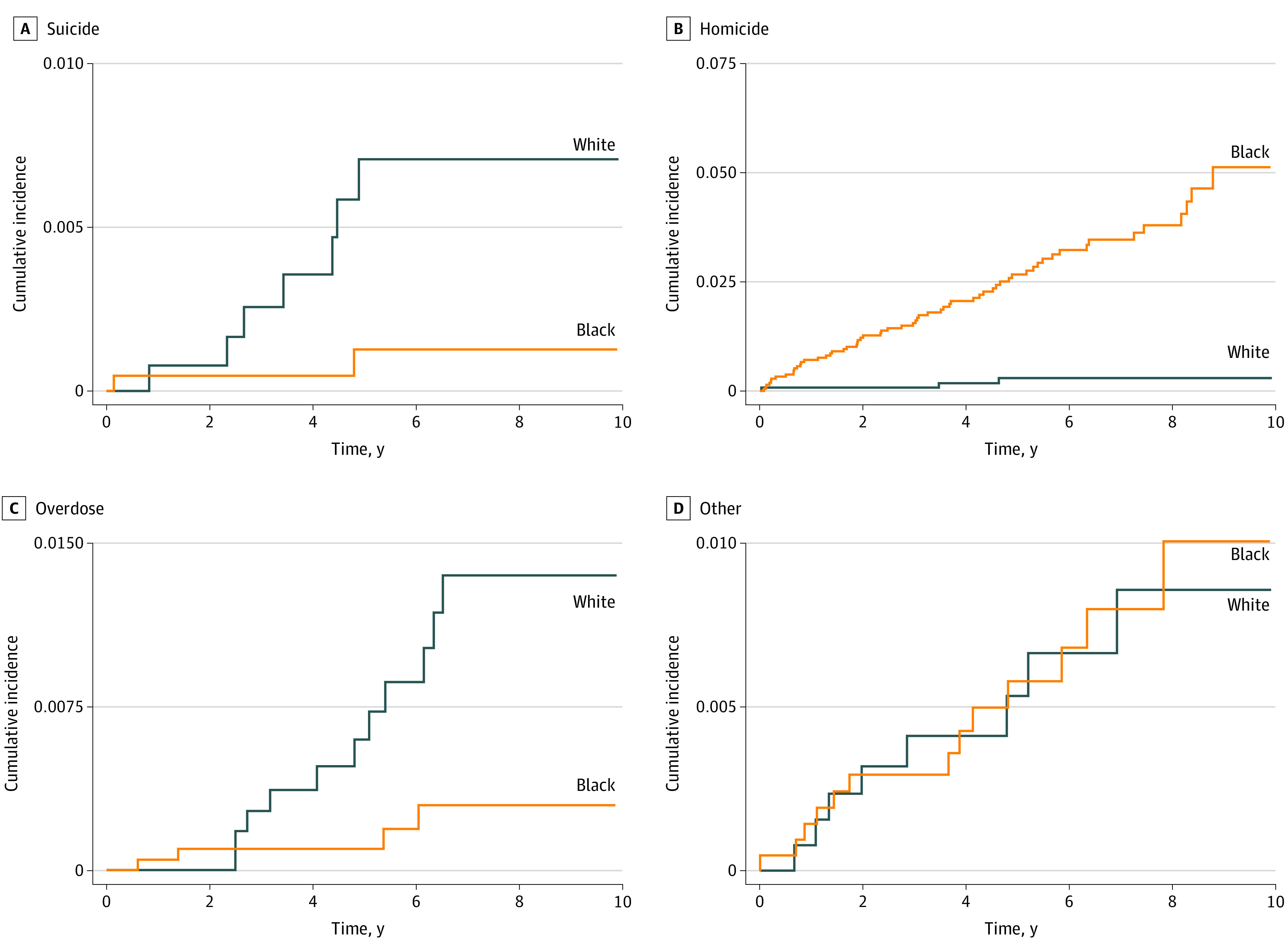
Cumulative Incidence by Cause of Death for Black and White Youths Previously Incarcerated in the Juvenile Legal System

The cumulative incidence by cause of death for previously incarcerated Black and White youths can be found in Figure 2. Black youths had more than 8 times higher 5-year cumulative incidence of homicide (2.67%) compared with White youths (0.32%). White youths had higher 5-year cumulative incidence of suicide (0.6% vs 0.1%) and overdose (0.5% vs 0.1%) compared with Black youths Cumulative incidence for other causes of death was similar.

## Discussion

Research examining early mortality among previously incarcerated youths has been limited, challenging our ability to address this public health issue. This study is the first, to our knowledge, to assess all-cause mortality rates and specific causes of death in youths with a history of incarceration in long-term juvenile correctional facilities and a comparable group of youths in the general population. Previously incarcerated youths were at an increased risk of early mortality, with notable differences among demographic subgroups. Most formerly incarcerated youth decedents were male, Black, younger than 21 years, and released from confinement between the ages of 18 and 21 years. More than half of all deaths were among youths convicted of crimes against persons. More deaths occurred in youths who were incarcerated for the first time and in youths who spent less than or equal to 1 year in custody. Homicide was the leading cause of death among previously incarcerated youth, accounting for more deaths than suicide, overdose, and other causes combined.

Of particular significance was the mortality rate in previously incarcerated females. Although females represented a considerably smaller number of youths who were incarcerated, the all-cause mortality rate among females was nearly 9 times that of the Medicaid comparison group. Teplin et al^8^ reported similar results, finding the mortality rate for previously detained females was 8 times the rate in the general population. Homicide was the most common cause of death for both males and females with a history of incarceration. Suicide rates were higher in males than females for previously incarcerated and Medicaid-enrolled youth, corroborating existing evidence that more males die by suicide than females.^16,17^ The absence of suicide as a cause of death among females who were incarcerated may be a consequence of the relatively small numbers in our sample.

Homicide was the leading cause of death among Black youths who were previously incarcerated and Medicaid-enrolled youth, with previously incarcerated Black youths significantly more likely to die by homicide than Black youths in the Medicaid comparison group. Although overdose was the most prevalent cause of death in formerly incarcerated White youth, a noteworthy finding was in mortality rates by suicide, which was 7 times that of Medicaid-enrolled youths. There were no reported deaths by suicide or overdose for youths of other race/ethnicity with a history of incarceration. Mortality risk was highest in previously incarcerated youths aged 15 to 21 years, suggesting that mortality risk may diminish with increasing age into young adulthood.

### Implications

Incarceration is a risk marker for early mortality in youth, and our study results suggest that successful strategies to prevent delinquency and reduce or even eliminate youth incarceration have the potential to decrease mortality risk in this vulnerable population. Effective approaches, such as counseling, mentoring programs, family-centered interventions, and school-based initiatives, have considerably decreased the number of youths who come in contact with the law.^2,18,19^ Similar services are also needed to ensure youths released from incarceration successfully integrate back into the community and to prevent youths from reengaging in delinquent behavior.^20,21,22^

Consistent with prior evidence,^6,7,8,9^ formerly incarcerated youths were significantly more likely to die by homicide than youths in the Medicaid comparison group. Strategies to decrease the risk of homicide following incarceration are worthy of exploration and call attention to homicide as a major cause of death in adolescents and young adults in general. More than 98% of homicides were by firearm, emphasizing the importance of interventions focused on firearm violence prevention, including educational programs,^23,24^ youth focused firearm laws,^25^ and safe firearm storage public awareness campaigns.^23,26^ An evidenced-based violence prevention program developed specifically for incarcerated youths is the BUILD Violence Intervention Curriculum, which aims to deter youths from engaging in violent firearm-related activities, such as gangs, drugs, and crime, when returning to their communities.^27,28,29^

Previously incarcerated youths can also benefit from suicide prevention strategies, including the potential utility of more robust mental health screening and suicide risk assessment in correctional settings.^30,31^ Empirically supported interventions for preventing delinquency and youth suicidal behavior, such as cognitive behavioral therapy, could provide encouraging solutions.^32,33,34,35^ Youths who were incarcerated also experienced a higher rate of death by overdose than Medicaid-enrolled youth, highlighting the importance of responding to the substance use disorder needs of previously incarcerated youths.^36,37^

Formerly incarcerated females were at a considerable higher risk of early mortality compared with female Medicaid-enrolled youths. Research shows complex histories of trauma contribute to females in the juvenile legal system to a much greater extent than males.^38,39,40^ Incarceration can exacerbate the adverse effects of these experiences, justifying a trauma-informed approach to delinquency prevention for females.^39,40^ An intervention with demonstrated efficacy for the treatment of trauma among females involved with the justice system is the Trauma Affect Regulation: Guide for Education and Therapy (TARGET) program.^41,42^ Drawing on a strengths-based model of care, TARGET promotes healthy coping skills to better manage reactions and emotions related to traumatic life events.^41,42^

Efforts to prevent juvenile delinquency have resulted in a 60% decline in youth incarceration rates in the past 20 years.^1^ Unfortunately, reducing the disproportionate representation of youths from minoritized racial and ethnic groups who are incarcerated has not followed the same trajectory.^1^ Nowhere is this inequity more pronounced than in Black youth, who account for 40% of all incarcerated youths in the United States but only 14% of the population younger than 18 years.^1^ Along with existing statistics, our finding that previously incarcerated Black youths also had the highest mortality rate underscores the need for more culturally relevant delinquency prevention strategies. Despite federal mandates and substantial investments in multistate initiatives to address racial and ethnic disproportionality in the juvenile legal system,^43,44,45^ a growing body of research has raised concerns about the effectiveness of these efforts and their ability to enact meaningful change.^45,46,47^ One promising approach is the Strong African American Families Program, developed for Black youths aged 10 to 14 years and their caregivers,^48,49^ which focuses on strengthening family relationships to help youths avoid problematic behaviors.^48,49^

Most previously incarcerated youths died before the age of 21 years, suggesting delinquency prevention strategies that consider the developmental needs of youths are necessary. School settings can play an important role in delinquency prevention by providing age-appropriate programs that potentially reach a large number of youths.^50^ Families and Schools Together focuses on building supportive relationships between caregivers and school personnel with proven effectiveness addressing disruptive behaviors and preventing delinquency in school-aged youths.^51,52^

Lastly, findings support the advancement of justice reform initiatives aimed at reducing and even eliminating youth incarceration. Community-based diversion and treatment-oriented programs that redirect youth offenders from the legal system are found to be more effective at preventing recidivism compared with the conventional prosecution process.^53,54,55^ Diversion programs based on restorative justice models are shown to be particularly promising. A meta-analysis found that youths participating in restorative diversion programs were more than 75% less likely to be rearrested in the year or 2 following diversion than court-processed youths.^56^

### Limitations

This study is not without limitations. First, the sample for this study is youths incarcerated in the Ohio juvenile legal system, and results may not be fully generalizable to other states. However, demographic characteristics of incarcerated youths in Ohio’s juvenile correctional facilities are aligned with those at the national level, and findings will likely be relevant.^1^ An exception is the higher representation of Hispanic and American Indian or Alaska Native incarcerated youths in the United States compared with Ohio, which reflects slightly higher numbers of Black youths.^1^ Second, youths in this study were enrolled in Medicaid, and findings may not be applicable to privately insured or uninsured youths. Results may also not apply to youths enrolled in other state Medicaid programs given differences in eligibility requirements and service options. Third, based on data restrictions, potential temporal trends in mortality rates were not accounted for in the analyses. Fourth, death certificate data commonly used to identify cause of death may be misclassified and underestimated.

## Conclusions

In this study, previously incarcerated youths were significantly more likely to experience early death compared with Medicaid-enrolled youths in the general population. Homicide was the leading cause of death among youths who were incarcerated, with mortality rates highest in those who were male, Black, and between the ages of 15 and 21 years. Formerly incarcerated females were also at heightened risk of all-cause mortality, homicide, and overdose relative to same-sex Medicaid-enrolled youths. Delinquency and violence prevention strategies that incorporate a culturally informed approach and take both sex and developmental level into consideration are critical.

## Notes

Supplement. **eTable.** *ICD-10* Causes of Death
