# Supplementary material for: Mortality and Cause of Death Among Youths Previously Incarcerated in the Juvenile Legal System
Source: JAMA Netw Open. 2021 Dec 23;4(12):e2140352. doi: 10.1001/jamanetworkopen.2021.40352 (PMC8703246; doi:10.1001/jamanetworkopen.2021.40352)
Supplement: Supplement. — eTable. ICD-10 Causes of Death [file jamanetwopen-e2140352-s001.pdf]

Supplemental Online Content

Ruch DA, Steelesmith DL, Brock G, et al. Mortality and cause of death among youth previously incarcerated in the juvenile legal system. *JAMA Netw Open*. 2021;4(12):e2140352. doi:10.1001/jamanetworkopen.2021.40352

eTable. *ICD-10* Causes of Death

This supplemental material has been provided by the authors to give readers additional information about their work.

© 2021 Ruch DA et al. *JAMA Network Open.*

eTable. *ICD-10* Causes of Death

Cause of Death	ICD 10 Codes
Homicide / Legal Intervention	X85-Y09, Y87.1, Y35.0
Overdose	X40-X44
Suicide	X60–X84, Y87.0, U03
Other	
Natural Causes	C81.9, C91.0, E14.1, F11.9, G40.9, I26.9, I38, I42.0, I42.9, I60.9, J18.0
Accident	V03.1, V47.5, V89.2, X00
Unknown/Undefined	R99, Y24

© 2021 Ruch DA et al. *JAMA Network Open.*
